# Moderate to severe renal impairment cannot be reliably detected from medical history alone: Implications for the use of Gadolinium-based contrast agents

**DOI:** 10.1186/1532-429X-11-S1-P123

**Published:** 2009-01-28

**Authors:** John-Paul Carpenter, Elizabeth Burman, Raad Mohiaddin

**Affiliations:** Royal Brompton and Harefield NHS Trust, London, UK

**Keywords:** Chronic Kidney Disease, Renal Impairment, Chronic Kidney Disease Stage, Severe Renal Impairment, Nephrogenic Systemic Fibrosis

## Introduction

Nephrogenic systemic fibrosis (NSF) is a rare condition characterised by thickening and tightening of the skin, hyperpigmentation and limitation of joint mobility. Autopsy data suggests that NSF may also be a more systemic disorder, affecting the heart, lungs, liver and other organs. An association with gadolinium-based contrast agents (GBCA) was first reported in 2006 [1] and therefore, the US Food and Drug Administration (FDA) recommends that GBCA 'should not be given in patients with known risks for developing NSF unless the diagnostic information is essential and cannot be obtained with non-contrast enhanced MRI or other diagnostic procedures'. The guidelines also state that patients should be evaluated for renal dysfunction either by obtaining a history or conducting laboratory tests [2]. Although NSF has only been reported in chronic kidney disease (CKD) patients with estimated glomerular filtration rate (eGFR) <30 ml/min/1.73 m^2^, it has been documented in acute renal failure patients with an intercurrent illness and eGFR of between 30 and 60 ml/min/1.73 m^2^ [3].

## Purpose

This study was designed to investigate whether patients with moderate to severe renal impairment could be easily identified from history alone or whether routine blood testing is required in every patient prior to the use of gadolinium-based contrast agents.

## Methods

Serum creatinine and eGFR is routinely measured prior to the use of gadolinium chelates in our unit. eGFR is calculated using the Modification of Diet in Renal Disease (MDRD) formula. As part of the pre-scan safety questionnaire, patients are asked if they are aware of any kidney problems.

Over a 3-month period from January 2008 to March 2008, all cases were reviewed to identify patients with moderate or severe renal impairment. This was defined as CKD Stage 3 (eGFR 30–60 ml/min/1.73 m^2^), Stage 4 (eGFR 15–30 ml/min/1.73 m^2^) or Stage 5 (End stage renal failure with eGFR <15 ml/min/1.73 m^2^). Documentation was then reviewed to see if renal impairment had been mentioned by the referring physician or was known by the patient themselves.

## Results

**Figure 1:**
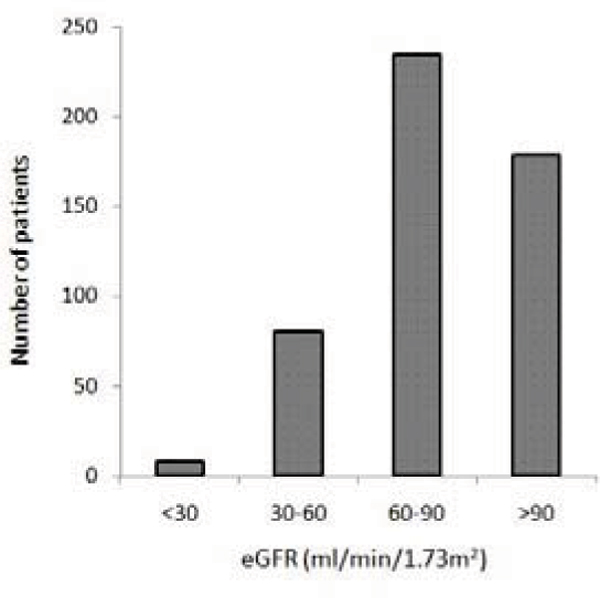
**Distribution of eGFR in patients referred for contrast-enhanced CMR scans**.

Of 944 scans performed in the three months from January 2008 to March 2008, 593 patients (62.8%) were given GBCA. 16% of patients with an indication for GBCA had an eGFR of 30–60 ml/min/1.73 m^2^ and 1.6% had an eGFR of <30 ml/min/1.73 m^2^. Only one patient had an eGFR of <15 ml/min/1.73 m^2^. The distribution is shown in Figure 1.

**Figure 2:**
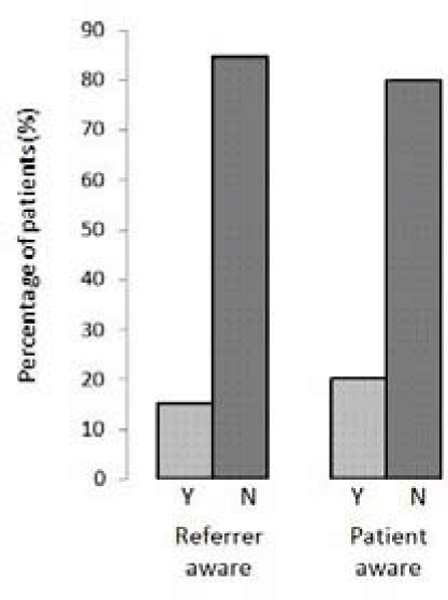
**Awareness of moderate to severe renal impairment (eGFR 30–60 ml/min/1.73 m**^**2**^**) prior to blood results at time of CMR scan**.

All patients with eGFR of <30 ml/min/1.73 m^2^ were identified either by the referring physician or by the safety questionnaire prior to the scan. The situation was very different for those with eGFR between 60 and 90. Of the 79 patients with eGFR in this range, only a minority were identified by the referring physician or were aware that they had renal impairment (15.2% and 20.3% respectively – Figure 2).

## Conclusion

This retrospective study highlights the significant numbers of patients with at least moderate renal impairment who are referred for CMR scans with an indication for GBCA. Our results suggest that most patients with moderate to severe renal impairment (eGFR 30–60 ml/min/1.73 m^2^) will not be identified without blood testing. In view of the fact that NSF has been reported in these patients (albeit with a concurrent illness), this has important implications and supports the current practice of measuring renal function on all patients. Decisions about giving gadolinium-based contrast agents need to be made based on clinical need for the information which will be gained together with the informed consent of the patient concerned.

## References

[1] Grobner T (2006). Gadolinium: a specific trigger for the development of nephrogenic fibrosing dermopathy and nephrogenic systemic fibrosis?. Nephrol Dial Transplant.

[2] [http://www.fda.gov/cder/drug/InfoSheets/HCP/gcca_200705.htm]

[3] Sadowski EA, Bennett LK, Chan MR (2007). Nephrogenic systemic fibrosis: risk factors and incidence estimation. Radiology.

